# Vancomycin-resistant *Enterococcus faecium* sequence type 796 - rapid international dissemination of a new epidemic clone

**DOI:** 10.1186/s13756-018-0335-z

**Published:** 2018-03-22

**Authors:** Andrew A. Mahony, Andrew H. Buultjens, Susan A. Ballard, Elizabeth A. Grabsch, Shirley Xie, Torsten Seemann, Rhonda L. Stuart, Despina Kotsanas, Allen Cheng, Helen Heffernan, Sally A. Roberts, Geoffrey W. Coombs, Narin Bak, John K. Ferguson, Glen C. Carter, Benjamin P. Howden, Timothy P. Stinear, Paul D. R. Johnson

**Affiliations:** 1 Department of Infectious Diseases, Austin Health, 145 Studley Rd, Heidelberg, VIC 3084 Australia; 2 Department of Medicine, The University of Melbourne, Heidelberg, VIC 3084 Australia; 3 Department of Microbiology and Immunology, The University of Melbourne at The Peter Doherty Institute for Infection and Immunity, Melbourne, VIC 3000 Australia; 4 Microbiological Diagnostic Unit Public Health Laboratory, The University of Melbourne at The Peter Doherty Institute for Infection and Immunity, Melbourne, VIC 3000 Australia; 5 Department of Microbiology, Austin Health, Heidelberg, VIC 3084 Australia; 6 Melbourne Bioinformatics, The University of Melbourne, Parkville, VIC 3010 Australia; 7 Monash Infectious Diseases, Monash Health, Clayton, VIC 3168 Australia; 8 Department of Infectious Diseases, Alfred Health, School of Public Health and Preventive Medicine, Monash University, Prahran, VIC 3181 Australia; 9 Antimicrobial Reference Laboratory, Institute of Environmental Science and Research (ESR), Wellington, 5022 New Zealand; 10 Department of Clinical Microbiology, Auckland District Health Board, Auckland, 1051 New Zealand; 11 School of Veterinary and Life Sciences, Murdoch University, Murdoch, WA 6150 Australia; 12 Microbiology Department, PathWest Laboratory Medicine – WA, Fiona Stanley Hospital, Murdoch, WA 6150 Australia; 13 Department of Infectious Diseases, Royal Adelaide Hospital, Adelaide, South Australia 5000 Australia; 14 Division of Microbiology, Health Pathology, NSW Department of Immunology and Infectious Diseases, John Hunter Hospital, University of Newcastle, Newcastle, NSW 2305 Australia

**Keywords:** VRE, Whole genome sequencing, Molecular epidemiology, Outbreak, Infection control

## Abstract

**Background:**

Vancomycin-resistant *Enterococcus faecium* (VRE) is a leading cause of hospital-acquired infections. New, presumably better-adapted strains of VRE appear unpredictably; it is uncertain how they spread despite improved infection control. We aimed to investigate the relatedness of a novel sequence type (ST) of *vanB E. faecium* - ST796 - very near its time of origin from hospitals in three Australian states and New Zealand.

**Methods:**

Following near-simultaneous outbreaks of ST796 in multiple institutions, we gathered then tested colonization and bloodstream infection isolates’ antimicrobial resistance (AMR) phenotypes, and phylogenomic relationships using whole genome sequencing (WGS). Patient meta-data was explored to trace the spread of ST796.

**Results:**

A novel clone of *vanB E. faecium* (ST796) was first detected at one Australian hospital in late 2011, then in two New Zealand hospitals linked by inter-hospital transfers from separate Melbourne hospitals. ST796 also appeared in hospitals in South Australia and New South Wales and was responsible for at least one major colonization outbreak in a Neonatal Intensive Care Unit without identifiable links between centers. No exceptional AMR was detected in the isolates. While WGS analysis showed very limited diversity at the core genome, consistent with recent emergence of the clone, clustering by institution was observed.

**Conclusions:**

Evolution of new *E. faecium* clones, followed by recognized or unrecognized movement of colonized individuals then rapid intra-institutional cross-transmission best explain the multi-center, multistate and international outbreak we observed.

**Electronic supplementary material:**

The online version of this article (10.1186/s13756-018-0335-z) contains supplementary material, which is available to authorized users.

## Background

*Enterococcus faecium* has become a major cause of healthcare-associated bloodstream infections (BSI) worldwide [1, 2]. It is often postulated that antibiotic resistance mechanisms in *E. faecium* explain this success in hospitals. However, molecular typing has now defined clear lineages of hospital-associated *E. faecium* strains, nearly all of which belong to clade A1 (also defined as multi-locus sequence type [MLST] clonal complex 17), that are distinct from animal and non-hospitalized human commensal *E. faecium* [3–6]. Some researchers argue that hospital-adapted *E. faecium* is sufficiently different to qualify as a separate species [7].

Vancomycin-resistant enterococcus (VRE) was first detected in Australasia in 1994, as *vanA E. faecium* in a clinical specimen from a liver transplant patient at Austin Health in Melbourne [8]. For the next 10 years, VRE colonization and infection remained uncommon in Australia and New Zealand. From 2005, several geographically separate hospitals in Australia noted increasing numbers of VRE colonized patients and VRE BSI caused by *E. faecium*. In contrast to the United States and Europe, these VRE have predominantly been *vanB E. faecium*, a phenomenon that may relate to *vanB* carriage within bowel anaerobes in the healthy population [9–11]. It was assumed that the sudden increase in detection was due to failures in infection control and cross transmission of existing endemic VRE clones. However, a detailed outbreak investigation at Austin Health documented the arrival in 2005 of a new sequence type of *E. faecium*, ST203 [12], initially as vancomycin-susceptible *E. faecium* (VSE), with subsequent acquisition of *vanB* determinants on multiple separate occasions in several distinct clones of ST203 VSE [13]. Results from national surveys demonstrated a similar sequence of events in other hospitals so that by 2011, ST203 VSE and VRE had become the most common cause of *E. faecium* BSI in hospitals across all Australian states [14]. Notably, in New Zealand VRE colonization and infection remained very uncommon.

Improved cleaning protocols were introduced at Austin Health following the ST203 VRE outbreak, which reduced the incidence of VRE BSI between 2009 and 2011 [15]. However despite ongoing enhanced cleaning, we once again observed an abrupt increase in *vanB* VRE *E. faecium* BSI from 2012 onwards that was caused by a novel ST – we lodged the alleles with the MLST Database (https://pubmlst.org/efaecium) and received the designation ST796 in September 2012 [16]. Although unknown prior to 2011, by 2013 *vanB* ST796 *E. faecium* had caused a large outbreak of colonization in a Melbourne Neonatal Intensive Care Unit (NICU) [17], and by 2014 was responsible for 66% of *E. faecium* VRE BSI in five separate Melbourne hospitals, largely replacing its ST203 predecessor [18].

To investigate the emergence of ST796 we established a multi-institutional working group to study the early history of ST796 *E. faecium* by whole genome sequencing (WGS). Our principal research question was whether this near-simultaneous multi-institution outbreak was best explained by repeated introductions from unrecognised VSE followed by independent acquisition of *vanB*, VRE community colonization, or by VRE cross-transmission in and between affected hospitals. We now describe the clinical impact, antibiotic resistance and phylogenetic relatedness of this newly emerged *E. faecium*.

## Methods

**Fig. 1:**
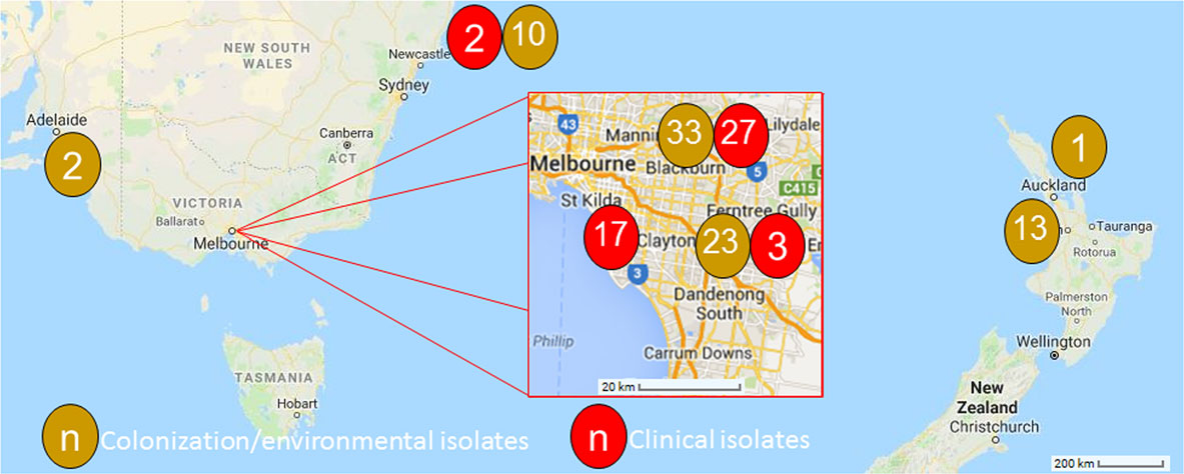
Source of 131 ST796 *E. faecium* isolates from seven hospitals across Australia and New Zealand

Working group participants from eastern Australia and northern New Zealand contributed ST796 *E. faecium* isolates from their local outbreaks – Fig. 1 shows the geographic distribution and number of isolates per institution. Initial identification of *E. faecium* was performed by the source hospital microbiology laboratory. Most isolates were originally classified as likely ST796 by Single Nucleotide Polymorphism High Resolution Melt (SNP HRM – in short, a rapid typing system based on the melt curves of common SNPs with variable G + C content) after referral to Austin Health [19]. Subsequently, speciation was re-confirmed by MALDI-TOF mass spectrometry or Vitek® 2 and the vancomycin resistance genotype determined by PCR as previously described [20]. A summary of the isolates with their epidemiological and meta-data is provided in Additional file 1: Table S1 and described in outline below.

### Rates of  *Enterococcus faecium* bacteremia at Austin health

Using discharge data collected by Austin Health’s information management system, rates of VRE *E. faecium* bacteremia by ST over 6-month periods per 1000 discharged patients were calculated from 2011 to 2014. These were compared with previously published rates from 1998 to 2010 [12].

### Antibiotic susceptibility testing

Antibiotic susceptibility testing was performed on all Austin Health, Alfred Health and John Hunter Hospital ST796 *E. faecium* blood culture isolates using the Vitek® 2 and Gram Positive Susceptibility card, AST-P612, according to the manufacturer’s instructions (bioMérieux). Austin Health isolates were further assessed by Etest® according to the manufacturer’s instructions (bioMérieux) for susceptibility to streptomycin, daptomycin, tigecycline, quinupristin-dalfopristin and chloramphenicol. Minimum Inhibitory Concentrations (MICs) were interpreted according to the Clinical and Laboratory Standards Institute (CLSI) guidelines where applicable.

### Provenance of 131 *Enterococcus faecium* isolates that underwent WGS

#### Alfred Health

Alfred Health, a university teaching hospital in Melbourne with solid organ and allogeneic bone marrow transplantation units, experienced a marked increase in VRE BSI in 2014; 17 consecutive VRE BSI isolates (all *E. faecium*) were investigated as the STs and relatedness of isolates were unknown. Sixteen isolates were found to be ST796 (the other was ST203). One BSI isolate from 2013 identified retrospectively via SNP HRM typing as likely to be ST796 was included in the analysis [21].

#### Auckland City Hospital

Thirteen isolates were referred from Auckland City Hospital, New Zealand, during a VRE colonization outbreak that affected more than 50 hospitalized patients in 2012. The outbreak appeared to be clonal by pulsed field gel electrophoresis (PFGE, data not shown) performed at the national reference laboratory (ESR, Wellington, New Zealand), and followed the repatriation in 2012 from Monash Health in Melbourne of a patient who had been hospitalized while travelling in Australia and acquired VRE prior to transfer. This patient was found to have two PFGE pulsotypes of colonizing VRE *E. faecium*, detected serendipitously on antibiotic susceptibility testing, with a double zone of inhibition observed around a streptomycin disc; one matched the outbreak ST796 by MLST, whereas the other pulsotype was ST203. The patient’s earlier rectal colonization and liver abscess VRE *E. faecium* isolates from Monash Health were identified as ST203.

A single colonizing isolate from a patient in another Auckland hospital, with no known links to Auckland City Hospital, also typed as ST796 by MLST so was included in WGS; this patient had similarly been transferred from a Melbourne hospital in 2012.

#### Austin Health

To capture the first appearance of ST796 in Austin BSI cases and to monitor its incidence compared to other STs over time, 79 consecutive *E. faecium* isolates obtained from all episodes of BSI at Austin Health from 1st January 2011 to 31st December 2014 were assessed. Thirty-nine were *vanB* VRE, and 24 of these ST796. Three additional isolates from early 2015 were subsequently identified as ST796, so were included in phenotypic and phlyogenomic analyses. Additionally, 32 colonizing isolates of *E. faecium* over the same period obtained from high-risk patients who are routinely screened for VRE rectal colonization, and one environmental isolate detected on surveillance, with matching SNP HRM genotype to the first ST796 BSI isolate, were included – one VSE, 30 *vanB*, one *vanA* and one with both *vanA* and *vanB*.

#### John Hunter Hospital

One hundred nine *E. faecium* blood culture and selected screening isolates from John Hunter Hospital in New South Wales (NSW) obtained between 2007 and 2015 were available for WGS. Of these we identified 12 ST796 isolates, all from 2015. These comprised one VSE and one VRE from adults with positive blood cultures, and ten screening isolates from a NICU colonization outbreak.

### Monash Health

A large outbreak of VRE colonization in the NICU and special care nurseries at Monash Health in Melbourne was recognized in October 2013, with VRE never previously detected in these wards [17]. WGS was performed on 26 isolates (two environmental swab isolates, one urine culture, one eye swab, one blood culture and 21 screening samples from more than 40 colonized neonates).

### Royal Adelaide Hospital

Two of five patient isolates from a VRE colonization outbreak in September 2013 at the Royal Adelaide Hospital in South Australia matched the SNP HRM type of ST796 so were included in WGS analysis.

### WGS and bioinformatic analyses

Short read sequencing libraries were generated from genomic DNA using the Illumina Nextera XT DNA sample preparation kit. Libraries were sequenced on the Illumina platform using either the MiSeq with 250-cycle paired end chemistry or the NextSeq 500 with 150-cycle paired end chemistry according to the manufacturer’s instructions.

Snippy v3.2 (https://github.com/tseemann/snippy) was used to map sequence reads against the fully assembled Ef_aus0233 genome (GenBank accession no. PRJEB14733), a representative of the ST796 lineage [22]. Single nucleotide polymorphisms (SNPs) in core genome positions were used to construct a Maximum Likelihood tree with FastTree v2.1.8 [23]. The tree was used as a guide for ClonalFrame v1.7 to infer regions of recombination [24]. As previously described, a robust and recombination-free tree was generated [22]. Tree branches with less than 70% bootstrap support (500 replicates) were collapsed. Pairwise core non-recombinogenic SNP differences between isolates were tabulated and visualized using a custom R-script (https://github.com/MDU-PHL/pairwise_snp_differences).

### Ethics approval

VRE isolates were collected and compared, along with rates of BSI, using non-identifying data as part of standard infection control procedures under appropriately constituted infection control committees at each institution.

## Results

### Bacteremia at Austin Health

**Fig. 2:**
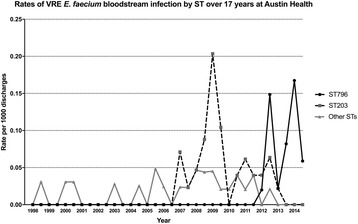
Rates of VRE *E. faecium* bacteremia by ST over 17 years at Austin Health, Melbourne. We have observed two recent outbreaks of VRE bacteremia, the first with ST203 which has now been almost completely replaced by ST796. *One isolate from 2010 was not typed

To better understand the changing epidemiology of *E. faecium* BSI at Austin Health, we plotted the rates at six-month intervals between 1998 and 2014 of VRE *E. faecium* BSI by ST (Fig. 2). This analysis showed the dominance of ST203 in 2009, then ST796 emerging in mid-2011 and becoming dominant by mid-2012. Of the 40 VSE (51% of all *E. faecium* BSI), nine were ampicillin-susceptible with all of these being community-associated infections, and none were ST796. Although ST796 and ST203 *E. faecium* caused BSI in roughly equal measure during 2012, the next 16 consecutive cases of VRE BSI over 15 months were all *vanB* ST796, almost completely replacing ST203.

### Phenotypic antibiotic resistance

Forty-six ST796 blood culture isolates underwent antimicrobial susceptibility testing (27 from Austin Health, 17 from Alfred Health, and two from John Hunter Hospital). All were ampicillin-resistant and all except one harboured *vanB*. Thirty-nine of 45 *vanB* isolates (87%) tested vancomycin-resistant, but two tested susceptible (MIC ≤4 mg/L) and four intermediate (MIC 8-16 mg/L). Thirty isolates (65%) had high-level gentamicin resistance. Two (4%) had intermediate susceptibility to linezolid (with none of the known 23S rRNA mutations or *optrA* genes; data not shown). All were susceptible to teicoplanin.

Additional testing of the Austin Health isolates revealed one with high-level streptomycin resistance, but no resistance to tigecycline, quinupristin-dalfopristin or chloramphenicol. All isolates were susceptible to daptomycin (although most had MICs of 3-4 mg/L). The ST796 isolates did not have additional antibiotic resistance or higher MICs than the ST203 clones they replaced (data not shown).

### Phylogenomics

**Fig. 3:**
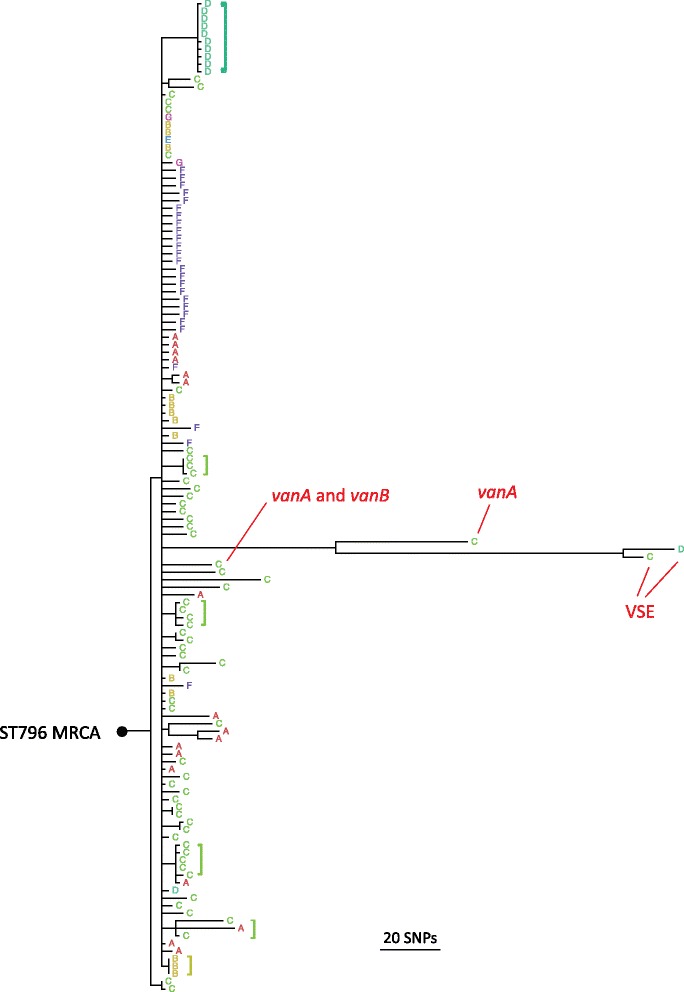
Maximum likelihood phylogenomic tree of 131 ST796 isolates. The tree was constructed from non-recombinogenic core SNPs and branches with less than 70% bootstrap support (500 replicates) were collapsed. The VSE, *vanA* and *vanB* isolates formed distinct and robust phylogroups. The single *vanA* + *vanB* isolate clustered within the *vanB* phylogroup. Branch lengths are proportional to core SNP differences with the scale as indicated. The inferred occurrences of intra-hospital evolution among the *vanB* isolates are labelled (brackets). The phylogenomic position of the ST796 most recent common ancestor (MRCA) in the ST796 tree was identified by using *Enterococcus hirae* (hirae_ATCC_9790, GenBank accession no. CP003504) as an outgroup. A = Alfred Health, Melbourne; B = Auckland City Hospital; C = Austin Health, Melbourne; D = John Hunter Hospital, Newcastle; E = other Auckland hospital; F = Monash Health, Melbourne; G = Royal Adelaide Hospital

The core genomes of 131 ST796 isolates were compared. SNPs were identified between these genomes and used to create a maximum likelihood phylogenomic tree (Fig. 3). To focus on the clonal heritage of the isolates, SNPs within regions of recombination (411 and 1895 clonal and recombinogenic core SNPs, respectively) were omitted from the sequence alignment that was used for tree building (Fig. 3).

The 128 *vanB* isolates formed a cohesive and monophyletic phylogroup (9.5 mean core SNPs) while the single *vanA* and two VSE ST796 isolates formed distinct phylogroups (20 core SNPs between the VSE isolates). The VSE phylogroup was furthest from the ST796 Most Recent Common Ancestor (MRCA) and was separated from the *vanA* and *vanB* phylogroups by 89 and 123 mean core SNPs, respectively. The single *vanA* isolate was separated from the VSE and *vanB* phylogroups by 89 and 61 mean core SNPs, respectively and was phylogenomically further from the ST796 MRCA than that of the *vanB* phylogroup.

The basal position of the *vanB* to that of the VSE isolates suggests that these isolates may have lost *vanB*. This feature of ST796 population structure was also observed in an accompanying study that focused on the genomic evolution of this emerging clone [22]. Similarly, this would also imply that the *vanA* isolate may have evolved from an ST796 progenitor that was originally *vanB*. One ST796 isolate with both *vanA* and *vanB* genotypes was identified; interestingly, this isolate clustered tightly within the *vanB* phylogroup, suggesting that it was originally harbouring *vanB* then horizontally acquired the *vanA* plasmid.

Despite the limited core SNP diversity observed among the *vanB* isolates, the existence of signature SNPs was linked to an isolate’s institution of origin. For some *vanB* isolates from a common institution there was sufficient core SNP identity to group them on a single node. The existence of isolates with identical core SNPs is indicative of sustained intra-institution circulation, where the founding bacteria have undergone a population bottleneck and consequently developed conserved alleles that are institution-specific. Despite such groupings, other *vanB* isolates formed singleton branches that radiate outward from the *vanB* MRCA. The singleton branches likely represent rapid evolutionary radiations from the *vanB* MRCA – a hallmark of a recently emerged and rapidly disseminated clone.

**Fig. 4:**
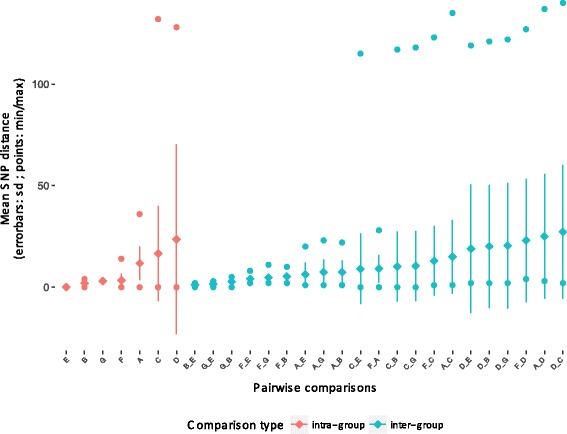
Pairwise comparisons of non-recombinogenic core SNP differences according to institution of origin. Overall, intra-hospital diversity is equal to or greater than inter-hospital diversity, indicating that there is substantial ST genomic admixture within each hospital. Comparisons are ordered according to increasing means. A = Alfred Health, Melbourne; B = Auckland City Hospital; C = Austin Health, Melbourne; D = John Hunter Hospital, Newcastle; E = other Auckland hospital; F = Monash Health, Melbourne; G = Royal Adelaide Hospital

To examine the relationship between the number of core SNP differences and the originating institutions, a pairwise core SNP comparison was undertaken (Fig. 4). The expectation for a clonal population is low intra-group diversity and higher inter-group diversity. Here, discrepancies between the intra-group and inter-group comparisons for all institutions were very modest and in some cases non-existent, indicating they do not contain single ST796 genotypes but rather multiple genotypes. Such substantial intra-institution diversity is consistent with a scenario of multiple inter-hospital transmission events.

## Discussion

*E. faecium* ST796 was first recognised at Austin Health at the beginning of 2012 and by 2014 had completely replaced the predecessor ST203 as a cause of BSI at that institution. This was not due to additional antibiotic resistance so the apparent enhanced fitness of ST796 is likely due to an alternative survival advantage. ST796 appeared almost simultaneously in two hospitals in Auckland New Zealand in 2012, and by 2015 had reached Newcastle, NSW. Using WGS to investigate this outbreak we observed genomic signals that were indicative of both rapid inter-hospital transmission and in some cases, sustained secondary intra-institution circulation. Our observation of a surge in BSI at Austin Health due to the appearance of ST796 is supported by results from Australian national surveys of *E. faecium* BSI from the Australian Group on Antimicrobial Resistance (AGAR). In the 2010 survey, ST796 was not detected, however in 2014, ST796 was responsible for 66% of BSI isolates in Victorian hospitals, 14% in Tasmania and 4% in South Australia [18, 25, 26]. Notably ST796 was not detected in other Australian states, even though we now know it had reached John Hunter Hospital in NSW as a colonizing and infecting strain. This very rapid spread within Victoria indicates high transmissibility of ST796, but may also reflect different infection control procedures at the time of inter-hospital transfer or greater mixing of patients between institutions compared with other states. Similarly, a recent Victorian outbreak of carbapenem-resistant *Enterobacteriaceae* is now understood to be linked to admission or transfer from a single center [27].

In addition to BSI, ST796 *E. faecium* also caused rapid, clinically silent outbreaks of colonization in several locations: two hospitals in New Zealand, NICUs at Monash Health, and John Hunter Hospital, more than 800 km away. It is likely that undetected transfer of VRE between patients preceded the sharp rise in BSI in Victoria. In New Zealand, ST796 colonization was shown to be linked to international transfer of patients from hospitals in Victoria. We did not identify epidemiological links between other Melbourne hospitals and those where ST796 was identified in South Australia or NSW. However it seems likely that ST796 was disseminated in a similar manner, carried by undetected colonized patients.

All *vanB* ST796 isolates we assessed are very closely related at the core genome level, irrespective of the location or date of isolation over three years. In contrast, significant differences in relationship were observed between the two ST796 VSE isolates and the ST796 *vanA* isolate when compared to their ST796 *vanB* counterparts (including the single *vanA* and *vanB* ST796 isolate). These differences are not explained by the presence or absence of different vancomycin resistance elements as these are excluded when determining core genome. Furthermore, all but one of the ST796 *E. faecium* isolated from blood cultures were vancomycin-resistant. This contrasts markedly with the previous ST203 outbreak at Austin Health and other mainland Australian states in which ST203 isolates were frequently clonally diverse and included VSE and VRE isolates [19], and with the complexity and diversity of hospital outbreak strains in a single center from Sydney [28].

This report has a number of limitations. Firstly, it is an observational study, with VRE screening not standardized across the institutions. Secondly, WGS was not performed on all new rectal colonization VRE isolates from the study sites due to cost, so we cannot assess the distribution of colonizing *E. faecium* STs, nor calculate the “attack rate” of ST796 from colonization to BSI. Thirdly, we cannot explain why the population structure of ST796 VRE has remained so clonal compared with the much greater diversity found within the previous ST203 outbreak strain. This suggests a survival advantage in hospitals of ST796 VRE over its predecessors but what exactly this is remains to be determined.

Cost, lack of single rooms and isolation fatigue make VRE control for those hospitals with endemic colonization a major challenge [21, 29]. Where VRE is not endemic, swift recognition and enhanced infection control may still be effective [17, 30]. Our data suggest that if we wish to control VRE colonization and infection in hospitals that are not yet endemic then more attention must be paid to movements of patients and possibly staff who have had contact with VRE-endemic hospitals.

## Conclusion

In conclusion, we have tracked a novel clone of ST796 *E. faecium* close to the start of its existence in Victoria Australia, where it has largely displaced ST203 as the leading cause of VRE BSI. In contrast ST796 has not caused significant invasive disease despite extensive colonization in a Melbourne NICU, John Hunter Hospital in NSW or in hospitals in Auckland – possibly due to differences in patients’ underlying medical conditions. WGS has demonstrated an essentially clonal population structure of ST796, but unlike the multi-drug-resistant gram-negatives, emerging healthcare-associated strains of *E. faecium* do not appear to depend for their success on enhanced antibiotic resistance. This suggests that other determinants of clonal success drive the rapid appearance and dissemination of new successful clones. One possibility is resistance to biocides rather than antibiotics per se; we are now actively investigating this possibility [31].

## Additional file

Additional file 1: **Table S1**. A summary of the isolates with their epidemiological and meta-data. (XLSX 22 kb)
